# Hearing Loss in Patients With Morquio Syndrome: Protocol for a Scoping Review

**DOI:** 10.2196/32986

**Published:** 2022-06-07

**Authors:** Lorena Diaz-Ordoñez, Estephania Candelo, Katherine Silva-Cuero, Wilmar Saldarriaga, Lenka Murgašová, Martin Magner, Harry Pachajoa

**Affiliations:** 1 Department of Basic Medical Sciences Center for Research on Congenital Anomalies and Rare Diseases (CIACER) Universidad Icesi Cali Colombia; 2 School of Basic Sciences Universidad del Valle Cali Colombia; 3 Centro de Investigaciones Clinicas Fundación Valle del Lili Cali Colombia; 4 Hospital Universitario del Valle Cali Colombia; 5 Department of Pediatrics and Inherited Metabolic Disorders General University Hospital and First Faculty of Medicine Charles University Prague Czech Republic; 6 Department of ENT University Hospital Kralovske Vinohrady and Third Faculty of Medicine Charles University Prague Czech Republic; 7 Department of Pediatrics Thomayer's University Hospital and First Faculty of Medicine Charles University Prague Czech Republic

**Keywords:** Morquio syndrome, hearing loss, rare diseases, scoping review

## Abstract

**Background:**

Mild to moderate hearing loss is common in patients with mucopolysaccharidosis (MPS) IVA. The hearing loss can be conductive, sensorineural, or mixed. However, in these patients, the mixed form is frequent, attributed to the combination of conductive and neurosensory elements, with slowly progressive evolution. Conductive hearing loss may be secondary to recurrent upper respiratory tract infections, serous otitis media, and deformities of the ear ossicles due to the accumulation of glycosaminoglycans (GAGs). Meanwhile, the sensorineural form is mainly attributed to the accumulation of GAGs in the auditory system.

**Objective:**

The aim of this scoping review is to understand the extent and type of evidence in relation to the physiopathology, classification, epidemiology, and clinical management of hearing loss and the effect of therapy for hearing loss in patients with MPS IVA.

**Methods:**

This scoping review includes participants across all genders and of no particular age group who are diagnosed with MPS IVA and develop hearing loss as a comorbidity. No exclusion criteria (country, language, or document type) will be applicable. The information sources will include experimental and quasi-experimental, analytical observational, observational, and qualitative studies. Unpublished literature will not be covered. Grey literature will be covered. A total of 2 independent reviewers will participate in the process of screening the literature, paper selection, and data extraction, and this process will be performed blindly. When all manuscripts have been selected, disagreements that arise between the 2 reviewers at each stage of the selection process will be resolved through discussion or with an additional reviewer. Results will be reported with descriptive statistics and information will be displayed in a diagrammatic or tabular manner, as explained in the JBI guidelines.

**Results:**

The literature search was performed in November 2021 in MEDLINE, LILACS (Literatura Latino-Americana e do Caribe em Ciências da Saúde), the Cochrane Library, ScienceDirect, Google Scholar, and OpenGrey; a total of 780 results were retrieved. Completion of the review is expected in mid-2022.

**Conclusions:**

This scoping review will be the first to describe the extent of the information regarding the development of hearing loss in the MPS IVA population. The data gathered by this review may lead to an understanding of the grade of hearing loss in this population and allow for the assessment of possible interventions according to the disease pattern.

**International Registered Report Identifier (IRRID):**

PRR1-10.2196/32986

## Introduction

### Background

Mucopolysaccharidosis (MPS) IVA, also known as Morquio syndrome, is a lysosomal storage disease caused by the loss of function of the enzyme N-acetylgalactosamine-6-sulfate sulfatase, which is required for the catabolism of glycosaminoglycans (GAGs), such as keratan sulfate and chondroitin 6-sulfate [1]. Biochemically, MPS IVA is characterized by an accumulation of GAGs within lysosomes and elevated GAG crystals in the urine, blood, and cerebrospinal fluid. GAG accumulation causes progressive damage in a wide variety of tissues, manifesting clinically as joint stiffness, bone malformation, growth retardation, restrictive lung disease, and liver, eye, heart, and dental abnormalities. Remarkably, patients with MPS IVA do not present primary neurological manifestations, although they can present behavioral problems, anxiety, and depression [2,3].

The life expectancy of patients with MPS IVA ranges from 8 to 43 years. Respiratory failure is the primary cause of death in patients with MPS IVA, followed by cardiac failure, posttraumatic organ failure, postoperative complications, and myocardial infarction [4]. Nevertheless, due to recent medical and surgical advances, the survival of these patients has improved. Hearing impairment is a common problem among patients with MPS IVA, and many of them exhibit permanent and severe hearing loss throughout life [5], so there is likely an increase in cases of moderate to severe hearing loss in the longest living patients [4]. In response, this scoping review will be conducted to obtain all published literature addressing the physiopathology, hearing loss classification, epidemiology, clinical management, and effect of therapy for hearing loss in patients with MPS IVA. A preliminary search of MEDLINE, the Cochrane Database of Systematic Reviews, and JBI Evidence Synthesis was conducted, and no completed or undergoing systematic reviews or scoping reviews on the topic were identified.

### Review Question

What is the current state of evidence on the physiopathology, classification, epidemiology, and management or treatment of hearing loss and the effect of therapy for hearing loss in patients with MPS IVA?

## Methods

The proposed scoping review will be conducted in accordance with the JBI methodology for scoping reviews [6].

### Eligibility Criteria

#### Participants

Studies that include participants of both genders and of no particular age who are diagnosed with MPS IVA and who developed hearing loss as a comorbidity will be selected.

#### Inclusion Criteria

The following studies will be selected for this review:

- Studies including patient hearing loss details, such as physiopathology, classification of hearing loss (neural, sensory, or sensorineural), and epidemiology (data on hearing loss in patients with MPS IVA according to age and gender)
- Studies providing information about the effects of replacement therapy on hearing loss in patients with MPS IVA
- Studies providing information about the medical management of patients with hearing loss due to MPS IVA and complications

#### Types of Sources

This scoping review will consider both experimental and quasi-experimental study designs including randomized controlled trials, nonrandomized controlled trials, before-and-after studies, and interrupted time series studies. In addition, observational studies including prospective and retrospective cohort studies, case-control studies, and analytical cross-sectional studies will be considered for inclusion. This review will also consider observational study designs, including case series, individual case reports, and descriptive cross-sectional studies for inclusion. Qualitative studies will also be considered for inclusion.

### Search Strategy

The search strategy will aim to identify published studies. Unpublished literature will not be included. The search strategy will focus on published studies without discriminating based on publication date or language. The key terms used in the article titles and abstracts as well as the articles’ index terms were used to develop a full search strategy for the following databases: MEDLINE, LILACS (Literatura Latino-Americana e do Caribe em Ciências da Saúde), Web of Science, the Cochrane Library, Trip Medical Database, Embase, ScienceDirect, and Google Scholar. Furthermore, a second search will be done to identify grey literature in OpenGrey and the Grey Literature Report.

To identify relevant articles, we established the following systematic search strategy using MeSH (Medical Subject Headings) keywords: “Hearing Disorders” OR “Hearing Loss” AND “Mucopolysaccharidosis IV” or “Hearing Disorders” OR “Hearing Loss” AND “Mucopolysaccharidosis IV”.

The search strategy, including all identified keywords and index terms, will be adapted for each included database and information source. Search terms will be created by the entire team of reviewers, though the search will be conducted by 1 person. The reference list of all included sources of evidence will be screened for additional studies. Studies published in any language will be included.

### Study Selection

All identified citations will be collated and uploaded into Rayyan QCRI23 (Rayyan Systems Inc) and duplicates will be removed. Titles and abstracts will then be screened by 3 independent reviewers for the application of the selection criteria. Then, the full text of the selected studies will be assessed in detail to verify inclusion. Reasons for the exclusion of studies at this stage that do not meet the inclusion criteria will be recorded and reported in the scoping review. Any disagreements that arise between the reviewers at each stage of the selection process will be resolved through discussion or with an additional reviewer. The results of the search and the study inclusion process will be reported in full in the final scoping review and presented in a PRISMA-ScR (Preferred Reporting Items for Systematic Reviews and Meta-analyses Extension for Scoping Reviews) flowchart [7].

### Data Extraction

A data extraction instrument was created (Multimedia Appendix 1). A total of 2 researchers will extract the data from each record. Extraction fields include the following: the country where the study was conducted, year of publication, data characterization (physiopathology, diagnostic, epidemiology, management or treatment, and complications), study design, medical history, clinical diagnosis, diagnostic test and results, and clinical interventions. Any disagreements that arise between the reviewers at each stage of the selection process will be resolved through discussion or with an additional reviewer(s).

### Data Analysis and Presentation

This will likely be a descriptive tabulation of all pertinent information regarding hearing loss in patients with MPS IVA, including physiopathology, risk factors, epidemiology, diagnosis, and prevention. Findings and recommendations, methods incorporated and their usefulness, and study limitations will also be captured. All knowledge gathered will be summarized and discussed.

### Reference Searches

Snowballing or citation tracking criteria will be used to identify important articles relevant to the topic of interest; this will also be done by using the reference list of a paper or citations of a paper by other articles to identify additional manuscripts relevant to the topic of the study. After identifying potential new manuscripts and citations, a backward snowballing search will be done by reviewing the reference list and excluding papers that do not fulfill the basic criteria for inclusion. Subsequently, papers from the list that have already been examined will be removed. If a paper meets the inclusion criteria, potential new manuscripts will be identified by checking the reference list of the included paper [8]. Following that, forward snowballing will be done by identifying new papers from the reference list of included papers with a similar approach as backward snowballing.

## Results

The search was performed on March 2, 2022, in MEDLINE, LILACS, the Cochrane Library, ScienceDirect, Google Scholar and OpenGrey; a total of 780 results were retrieved. Completion of the review is expected in mid-2022. All potential manuscripts will be imported into the reference management software Rayyan QCRI23. The results of this scoping review will identify and describe the epidemiology, physiopathology, classification, and clinical management of hearing loss in patients with Morquio syndrome. Additionally, it will also identify the known available effect of the current therapies (enzyme replacement therapy [ERT] and hematopoietic stem cell transplantation [HSCT]) on hearing loss in patients with MPS IVA.

## Discussion

The clinical spectrum of hearing loss in MPS IVA is wide and not well described yet. There has been limited research on the audiological assessment of patients with MPS IVA. However, some studies have suggested that the prevalence of hearing loss in this population varies between 67% to 94% [9-11]. According to this research, hearing loss can present in different forms, conductive, sensorineural, or mixed, and its severity can vary from mild to profound hearing loss. Furthermore, MPS IVA presents with recurrent otitis media [5,11]. Similar to other disorders, conductive hearing loss occurs at an early age, while sensorineural or mixed hearing loss develops later on in life [11]. The conductive component of hearing loss is likely caused by the synergistic presentation of recurrent otitis media and accumulation of GAGs on the tympanic membrane and the ossicular chain [5]. The physiopathology of sensorineural hearing loss is unknown, but a few studies have identified the possibility of hair cell loss contributing to sensorineural hearing loss, and animal models have described the role of the accumulation of keratan sulphate in the inner ear [5,12].

The efficacy of ERT on the audiological disorder of MPS IVA is still unknown, though a case report described an improvement with ERT therapy [13]. However, further studies with larger samples are needed to determine the efficacy of ERT on hearing improvement [14]. On the other hand, the role of HSCT therapy in treating hearing loss has not yet been determined [15-17]. A recent study has demonstrated the effect of cochlear implants on the improvement of postlingual hearing loss [18].

## Appendix

Multimedia Appendix 1 Data extraction instrument.

## Abbreviations

ERT: enzyme replacement therapy
GAGs: glycosaminoglycans
HSCT: hematopoietic stem cell transplantation
LILACS: Literatura Latino-Americana e do Caribe em Ciências da Saúde
MeSH: Medical Subject Headings
MPS IVA: mucopolysaccharidosis IVA
PRISMA-ScR: Preferred Reporting Items for Systematic Reviews and Meta-analyses Extension for Scoping Reviews
